# Patterns of Communicating the Diagnosis of Type 1 vs Type 2 Myocardial Infarction

**DOI:** 10.1016/j.jacadv.2026.102834

**Published:** 2026-06-17

**Authors:** Elaine Luterstein, Nhi Tong, Sean Murphy, Charlotte Paquette, Yuxi Liu, Jason H. Wasfy, Dalane Kitzman, John A. Spertus, Pradeep Natarajan, Cian P. McCarthy

**Affiliations:** a Department of Medicine, Mass General Brigham, Boston, Massachusetts, USA; b Harvard Medical School, Boston, Massachusetts, USA; c School of Pharmacy, University of California, San Francisco, California, USA; d Heart and Vascular Institute, Mass General Brigham, Harvard Medical School, Boston, Massachusetts, USA; e Wake Forest University School of Medicine, Winston-Salem, North Carolina, USA; f University of Missouri-Kansas City’s Healthcare Institute for Innovations in Quality and Saint Luke's Mid America Heart Institute, Kansas City, Missouri, USA; g Program in Medical and Population Genetics, Broad Institute of Harvard and MIT, Cambridge, Massachusetts, USA; h Baim Institute for Clinical Research, Boston, Massachusetts, USA

**Keywords:** communication, diagnosis, type 1 myocardial infarction, type 2 myocardial infarction

**Figure:**
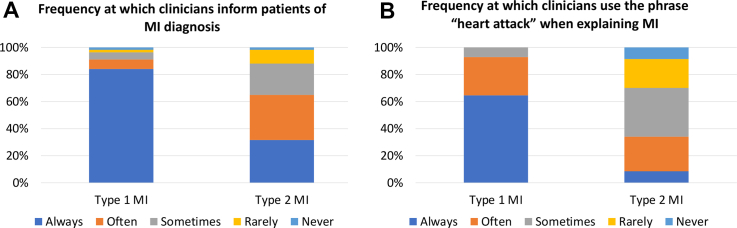

**What is the clinical question being asked?**

Are there differences in the communication of type 1 vs type 2 MI diagnoses to patients?

**What is the main finding?**

Clinicians report less frequently informing type 2 MI patients of their diagnosis and type 2 MI patients less often self-report a “heart attack” diagnosis.

Myocardial infarction (MI) is one of the most common inpatient cardiovascular diagnoses.^1^ Communicating the diagnosis—often described as a “heart attack”—is important for patient understanding of cardiovascular health and management.

Of the five MI subtypes, type 1 MI (T1MI) and type 2 MI (T2MI) are the most common and confer a high risk of recurrent cardiovascular events.^2^ However, in patient education materials, “heart attack” is often depicted as plaque rupture T1MI, while T2MI is infrequently mentioned.^3^ Furthermore, the diagnosis of T2MI can be ambiguous, with studies demonstrating only moderate diagnostic agreement among clinicians.^4^ To better understand this, we surveyed clinicians and patients within a large health care system to elucidate differences in the communication of MI subtypes.

## Methods

All study procedures were approved by the Mass General Brigham Institutional Review Board.

### Clinician survey

Faculty, trainees, and advanced practice providers from 5 Mass General Brigham hospitals identified from distribution lists for medicine, cardiology, and cardiothoracic/vascular surgery were invited by email (March 2025) to complete an electronic survey if they had cared for a patient with T1MI or T2MI. The survey, pilot tested among 10 clinicians, included 2 distinct questions: “In general, when a type 1 MI is diagnosed, how often do you inform the patient they have had type 1 MI?” and “When explaining to patients that they have had a type 1 MI, how often do you describe it as a 'heart attack'?” The questions were repeated for T2MI. The survey included standardized definitions of T1MI and T2MI (4th Universal Definition of MI).

### Patient survey

This single-center prospective study at Massachusetts General Hospital (October 2023–February 2025) enrolled consecutive inpatients with T1MI and T2MI until 150 patients (75 per MI subtype) were included. Patients lacking capacity to consent, pregnant, incarcerated, severely anemic, with active malignancy, hemodynamically unstable, receiving palliative care, or with another MI subtype within 6 months were excluded. If informed consent was obtained, participants completed questionnaires during their hospitalization and again by telephone, at 1 (4-6 weeks) and 4 months (16-18 weeks) after discharge. One of the questionnaires, the FRAIL Scale, included the question “Did your doctor ever tell you that you have had a heart attack?”, which was used for this analysis.

### Study outcomes and data analyses

The study outcomes were 1) the frequency at which clinicians report informing affected patients of their MI diagnosis and use the phrase “heart attack”; and 2) the proportion of patients who self-report a “heart attack” diagnosis on any of their questionnaires. Chi-square test was used to compare binary outcomes (“always”/“often” vs other responses) and chi-square trend test to compare ordinal variables. Multivariable logistic regression was used to assess: 1) whether clinician characteristics (sex, type, years of practice) were associated with “always” or “often” informing T2MI patients of their diagnosis; and 2) the odds of patients self-reporting a heart attack (adjusted for age, sex, race, prior MI, and MI subtype). Statistical analyses were performed using R software, version 4.2.2 (R Foundation for Statistical Computing).

## Results

### Clinician survey

A total of 2,250 potentially eligible clinicians received an email outlining the study and eligibility criteria, and 118 clinicians completed the survey. Among the respondents, 63 (53.4%) were female and 66 (55.9%) specialized in cardiology, 37 (31.4%) in internal medicine, and 4 (3.4%) in surgery. Respondents included 65 attendings (55.1%), 30 advanced practice providers (25.4%), and 23 trainees (19.5%); 67 (56.8%) had 10 or more years in practice. Most cared for both MI subtypes (112 [94.9%]).

**Figure 1:**
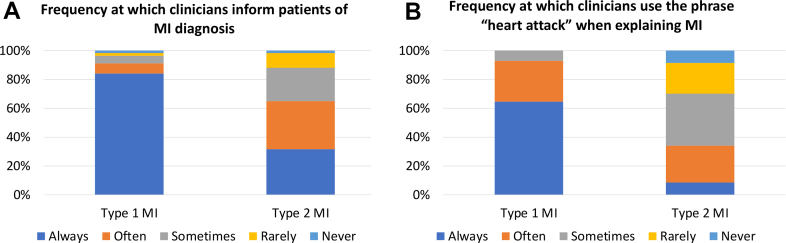
Clinician Communication of Myocardial Infarction Diagnosis Stratified By Subtype (A) Bar chart illustrating frequency at which clinicians report they inform myocardial infarction patients of their diagnosis stratified by subtype of myocardial infarction. (B) Bar chart illustrating frequency at which clinicians utilize the phrase “heart attack” when explaining the diagnosis of myocardial infarction stratified by subtype of myocardial infarction. MI = myocardial infarction.

Clinicians reported less frequently informing patients with T2MI of their diagnosis compared to T1MI (*P* < 0.001) (Figure 1A). Fewer clinicians reported “always” or “often” informing T2MI patients of their diagnosis compared to T1MI (76/117 [65.0%] vs 103/113 [91.2%], respectively, *P* < 0.001). Female clinicians more routinely “always” or “often” inform T2MI patients of their diagnosis (79.0% vs 49.1%; adjusted odds ratio: 3.75 [95% CI: 1.49-10.05]).

Clinicians less frequently use the phrase “heart attack” when communicating a diagnosis of T2MI compared with T1MI (*P* < 0.001) (Figure 1B). They less commonly “always” or “often” use the phrase “heart attack” when explaining a T2MI diagnosis compared with T1MI (40/117 [34.2%] vs 105/113 [92.9%], respectively, *P* < 0.001). Clinicians in practice for longer durations more frequently use the phrase “heart attack” when explaining T2MI.

### Patient survey

Over 16 months, 150 patients enrolled, but 1 participant withdrew from the data analysis, resulting in a final cohort of 74 with T1MI and 75 with T2MI; all completed their index questionnaire and 92 (61.7%) completed their 1- and 4-month questionnaires. The average age of participants was 67.6 ± 11.69 years, and 37 (24.8%) were female.

Among participants, 114/149 (76.5%) reported on any of their questionnaires being informed by their doctor that they had a “heart attack,” which was significantly less common in those experiencing a T2MI as compared with T1MI (61.3% vs 91.9%; *P* < 0.001). T2MI patients who underwent echocardiogram (93.9% vs 80.0%; *P* = 0.03) and invasive angiography during their index hospitalization (85.1% vs 34.4%; *P* < 0.001) more frequently reported being informed. In multivariable logistic regression analysis, individuals with T2MI were significantly less likely to report ever being informed by their doctor that they had a “heart attack” (adjusted OR: 0.13 [95% CI: 0.04-0.36]). Patients with MI who reported being informed they had a “heart attack” were more commonly prescribed aspirin (92.1% vs 62.9%; *P* < 0.001) and P2Y_12_ inhibitors (74.6% vs 22.9%; *P* < 0.001) at hospital discharge.

## Discussion

In a survey of diverse clinician types from several specialties within a large hospital network, respondents stated they more selectively inform T2MI patients of their MI diagnosis compared with T1MI. These data suggest some T2MI patients may be unaware of their diagnosis, which could potentially reduce risk-modifying opportunities. Selective communication may reflect diagnostic uncertainty,^4^ potential reclassification, lack of guideline-specified treatments, or comorbidities influencing prognosis.

Only one-third of clinicians report routinely using the phrase “heart attack” when communicating a T2MI diagnosis, and T2MI patients were almost 8 times less likely to report being informed by their doctor that they had a “heart attack.” These findings suggest that epidemiology analyses that utilize “heart attack” patient self-reporting may underestimate MI frequency. For example, national estimates of MI prevalence often rely on patient self-report.^1^ Patients were not explicitly asked whether they had been informed by their doctor that they had an “MI,” therefore, responses may reflect a mix of clinician terminology, patient interpretation, and recall. Clinicians may use the phrase “heart attack” less often to avoid confusion, as it is often depicted as a spontaneous event in patient education materials and media.^3^^,^^5^

Standardized language may help ensure patients understand a T2MI diagnosis, but caution is needed to avoid over-labeling, which could cause confusion or under-communication, which could leave patients unaware of their cardiovascular risk. Developing clear messaging may improve informed decision-making; however, further research is needed to determine the most effective approach.

The single-system study may limit generalizability. The study had a 5% clinician survey response rate, introducing potential nonresponse bias, and recall bias may have influenced both surveys. Cognitive status and health literacy were not assessed, which could affect patient comprehension and recall.

## Conclusions

The data from this analysis suggest some patients with T2MI may be unaware of their diagnosis and such gaps could be associated with missed opportunities for secondary prevention, warranting further investigation. Efforts toward improving communication regarding T2MI are needed.

## Funding support and author disclosures

Dr McCarthy is supported by a 10.13039/100000050National Heart, Lung, and Blood Institute under Award Number K23HL167659. The content of this manuscript is solely the responsibility of the authors and does not necessarily represent the official views of the 10.13039/100000002National Institutes of Health. Dr Natarajan has received research grants from Allelica, Amgen, Apple, 10.13039/100008497Boston Scientific, Cleerly, Genentech/Roche, Ionis, Novartis, and Silence Therapeutics; has received personal fees from AIRNA, Allelica, Apple, AstraZeneca, Bain Capital, Blackstone Life Sciences, Bristol Myers Squibb, Creative Education Concepts, CRISPR Therapeutics, Eli Lilly & Co, Esperion Therapeutics, Foresite Capital, Foresite Labs, Genentech/Roche, GV, HeartFlow, Incyte, Magnet Biomedicine, Merck, Novartis, Novo Nordisk, TenSixteen Bio, and Tourmaline Bio; has equity in Bolt, Candela, Mercury, MyOme, Parameter Health, Preciseli, and TenSixteen Bio; has received royalties from Recora for intensive cardiac rehabilitation; and has spousal employment at Vertex Pharmaceuticals, all unrelated to the present work. Dr Kitzman has received reimbursement as a consultant for and his institution has received reimbursement for clinical trials from Novo Nordisk, Bayer, and Rivus; has served as a consultant for Lilly and Boehringer Ingleheim; and holds stock in Gilead. Dr Spertus has provided consultative services on patient-reported outcomes and evidence evaluation to BioHaven, Janssen, Bristol Meyers Squibb, Terumo, Cytokinetics, BridgeBio, VentricHealth, and Imbria; holds research grants from the 10.13039/100000002National Institutes of Health, the 10.13039/100006093Patient-Centered Outcomes Research Institute, the 10.13039/100005485American College of Cardiology Foundation, Lexicon, Imbria, and Janssen; owns the copyright to the Seattle Angina Questionnaire, Kansas City Cardiomyopathy Questionnaire, and Peripheral Artery Questionnaire; and serves on the Board of Directors for Blue Cross Blue Shield of Kansas City. Dr McCarthy has received consulting fees or honorarium from 10.13039/100001316Abbott Laboratories, 10.13039/100006400Alnylam Pharmaceuticals, Azurity Pharmaceuticals, Inc, BioMerieux, Inc, Corcept Therapeutics, Cleerly Health, Heartflow, NewAmsterdam Pharma, and Roche Diagnostics. Dr Wasfy has been supported by an interim deliberative support award from the Executive Committee on Research at the Massachusetts General Hospital. All other authors have reported that they have no relationships relevant to the contents of this paper to disclose.
